# Whole genome sequencing data of a clinical *Enterococcus gallinarum* strain EGR748 from Sabah, Malaysia

**DOI:** 10.1016/j.dib.2020.106370

**Published:** 2020-10-02

**Authors:** Nur Nashyiroh Mastor, Vijay Kumar Subbiah, Wan Nazirah Wan Abu Bakar, Khurshida Begum, M. Jahangir Alam, Mohammad Zahirul Hoque

**Affiliations:** a Department of Pathobiology & Medical Diagnostics, Faculty of Medicine and Health Sciences, University Malaysia Sabah, Sabah, Malaysia; b Biotechnology Research Institute, University Malaysia Sabah, Sabah, Malaysia; c Department of Pathology, Queen Elizabeth Hospital, Kota Kinabalu, Sabah, Malaysia; d Department of Pharmacy Practice and Translational Research, University of Houston College of Pharmacy, Houston, TX, United States

**Keywords:** *Enterococcus gallinarum*, Whole genome sequencing, Antibiotic sensitivity and resistance, Sabah, Borneo

## Abstract

*Enterococcus gallinarum* is a gram positive facultatively anaerobic bacteria that is typically found in mammalian intestinal tracts. It is generally not considered pathogenic to humans and is rarely reported. Here, we present the draft genome sequence data of *Enterococcus gallinarum* strain EGR748 isolated from a human clinical sample, and sequenced using the Illumina HiSeq 4000 system. The estimated whole genome size of the strain was 3,730,000 bp with a *G* + *C* content of 40.43%. The *de novo* assembly of the genome generated 55 contigs with an N50 of 208,509 bp. In addition, the Maximum Likelihood phylogenetic analysis based on the 16S rRNA sequence data accurately clustered EGR748 with other *E. gallinarum* strains. The data may be useful to demonstrate the capacity of this enterococcal species becoming the causal agents of nosocomial blood-stream infections. The genome dataset has been deposited at DDBJ/ENA/GenBank under the accession number JAABOR000000000.

## Specifications Table

**Table:** 

Subject area	Microbiology
Specific subject area	Genomic. Antimicrobial resistance
Type of data	Genomic sequence. Tables and Figures
Data acquisition	Whole genome was sequenced, with an Illumina HiSeq 4000 system.
Data format	Raw and analyzed.
Parameters for data collection	*Enterococcus gallinarum* was isolated from the blood of a patient
Description of data collection	Strain EGR748 was sequenced with Illumina HiSeq 4000 platform and phylogenetic tree was then constructed based on the 16S rRNA region of 23 Enterococcus strains obtained from GenBank. The phylogeny indicated that this strain belonged to *Enterococcus gallinarum.*
Data source location	Queen Elizabeth Hospital, Kota Kinabalu, Sabah, Malaysia
Data accessibility	Data is publicly available at DDBJ/ENA/GenBank under the accession number JAABOR000000000; (www.ncbi.nlm.nih.gov/nuccore/JAABOR000000000); the version described in this paper is version JAABOR010000000.(Bioproject/ PRJNA601613)

## Value of the Data

• The draft genome data of *Enterococcus gallinarum* strain EGR748 highlights clinically important rare non-*faecalis* and non-*faecium Enterococcus* obtained from the blood of human patient.
• The antibiotic sensitivity and resistance data of *Enterococcus gallinarum* will be useful for clinicians in empirical treatment strategies especially in treating enterococcal bloodstream infection.
• The data will help the researcher to understand the emergence of rare *Enterococcus gallinarum* in the clinical setting and whether it falls in homogenous group with the common human enterococcal infection, *Enterococcus faecalis* and *Enterococcus faecium.*

## 1 Data Description

**Fig. 1:**
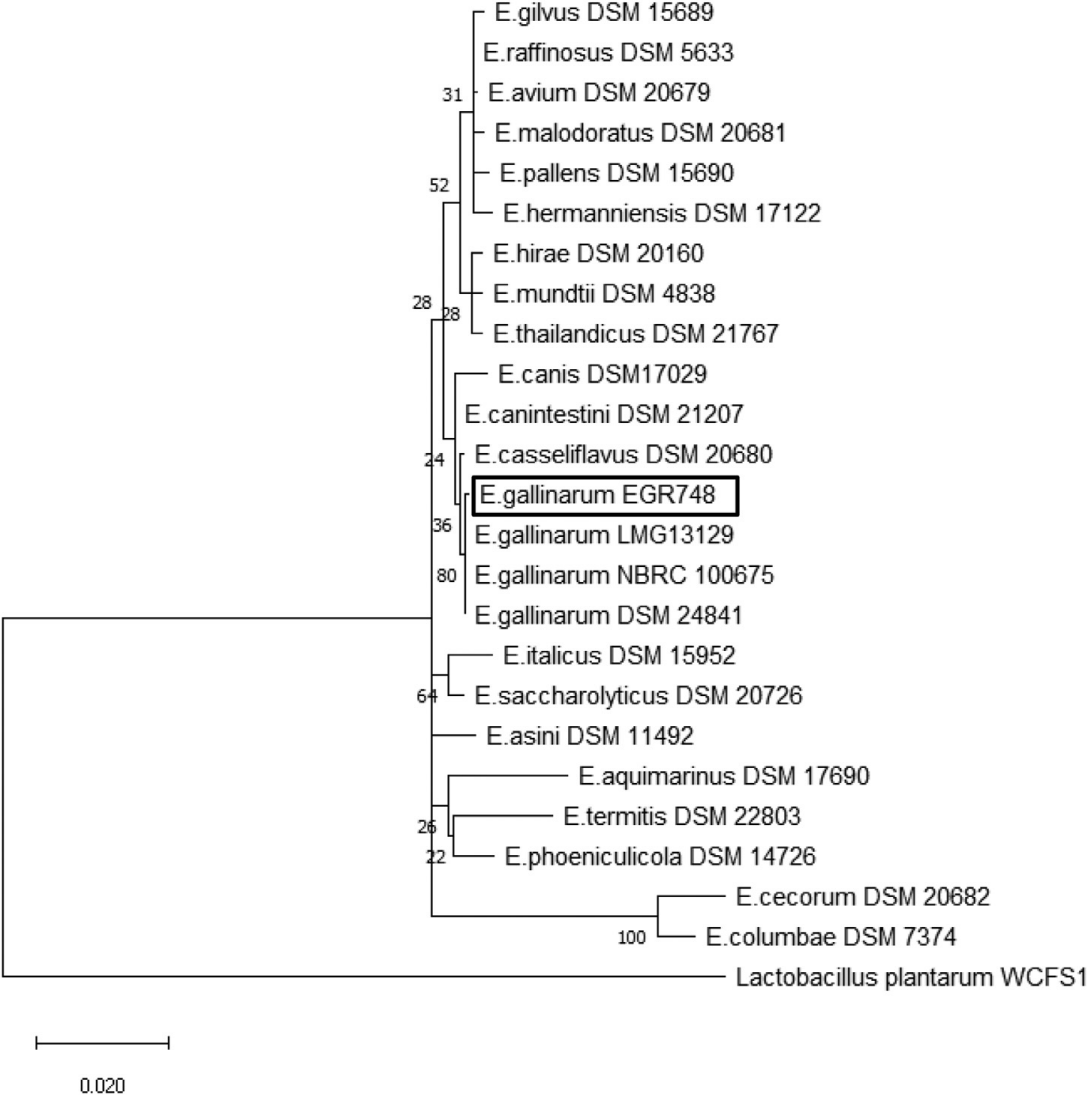
Phylogenetic tree showing the position of *Enterococcus gallinarum* strain EGR748 based on 16S rRNA sequences with other 23 *Enterococcus* species. The tree was constructed using MEGA 6.0 with 1000 bootstrap iterations.

*Enterococcus gallinarum* is rarely associated with clinical infections in humans and is not widely reported as compared to its closely related common nosocomial pathogen such as, *Enterococcus faecalis* and *Enterococcus faecium.* In this study, we present the whole genome sequence data of *Enterococcus gallinarum* strain EGR748 which was isolated from the blood of a 63-years old male in August 2019. The patient was admitted to the Queen Elizabeth Hospital at Kota Kinabalu, Sabah, with symptoms of tuberculosis and septic shock. A total of 10,488,498 paired reads of a 300-bp insert-size library by NEBnext Ultra kit (New England Biolabs, NEB #E7645) were generated from the Illumina HiSeq 4000 and *de novo* assembly of the genome generated 55 contigs with N50 of 208,509 bp (Table 1). The whole genome size was 3730,000 bp with a *G* + *C* content of 40.43% and consistent with the findings of a previous report [1]. In addition, the phylogenetic tree from 16S rRNA sequence data which was obtained separately from Sanger Sequencing (Supplementary Data), accurately placed strain EGR748 with other *Enterococcus gallinarum* strains (Fig. 1). The screening of antimicrobial resistance gene in *Enterococcus gallinarum* strain EGR748 by using ResFinder version 1.3 revealed that this strain was found to be resistant to macrolide (erm(B), glycopeptide (VanC1XY) and aminoglycoside ((ant(6)-la and aph(3′)-III)). Finally, annotation using RAST (Rapid Annotation of microbial genome using Subsystem Technology) showed 255 subsystems, 3342 coding sequences and 60 RNA genes**.** Analysis of the genomes of the *Enterococcus gallinarum* will increase our insight into the potential adaptation mechanism of this uncommon *Enterococcus* species in clinical settings and the factors of resistance gene mobility that could influence the host-microbe interactions.

**Table 1:** Statistics of assembled sequence of *Enterococcus gallinarum* EGR748.

ASSEMBLY STATISTIC	FINAL ASEMBLY
Total sequence read	10,488,498
Sequencing Depth	305x
No of contigs	55
Total sequence length (bp)	3306,813
Minimum sequence length (bp)	226
Maximum sequence length (bp)	753,431
N50(bp)	208,509
GC%	40.43%
CDSs	3142
tRNAs	56
5 s,16 s,23 s rRNA	1,1,2

## 2 Experimental Design, Materials and Methods

### 2.1 Isolation, culture, DNA extraction, library preparation and sequencing

The *Enterococcus gallinarum* strain EGR748 was isolated from a patient's blood and the strain was grown overnight at 37 °C on blood agar and further sub-cultured on Luria Bertani (LB) broth High Salt (MB cell). The identification of species was initially done using fully automated VITEK2 (BioMérieux, Inc, Hazelwood, Mo) system and subsequently confirmed by 16S rRNA Sequencing using the BigDye Terminator (v3.1) on a ABI3130 Sequencer. Genomic DNA was extracted using Qiagen DNeasy kit (Qiagen, Valencia, CA) according to the manufacturer's protocol. The measurement of DNA concentration was determined by Nanodrop 2000c spectrophotometer (ThermoFisher Scientific, USA) and Qubit® 2.0 fluorometer (Invitrogen, ThermoFisher Scientific, USA). Whole genome sequencing libraries were prepared using the NEBnext Ultra kit (Illumina, San Diego, CA) and sequenced with the Illumina HiSeq 4000 platform.

### 2.2 Quality assessment, *de novo* assembly, gene annotation and screening of antimicrobial resistance gene

The genome sequencing coverage performed was 305x and sequenced until 99% completion. The data sequence was deposited in the Sequence Read Archive (SRA) (Biosample accession number SAMN13870908) under the bioproject accession number PRJNA601613. For the purpose of analysis, the sequence read quality was checked using FastQC. All of the raw reads were pre-processed, the adapters were trimmed and the reads with less than 50 bp were removed, based on phred with a quality below Q30 using Trimmomatic version 0.39 [2]. The genome was assembled using IDBA-UD (iterative de Bruijn graph assembler) [3] and annotated with RAST (Rapid Annotation of using System Technology) [4] and antimicrobial resistance gene was detected using ResFinder v3.0 [5].

### 2.3 Phylogenetic analysis of *Enterococcus gallinarum* strain EGR748

A comparative sequence analysis of the 16S rRNA gene was performed to obtain a phylogenetic tree and to observe the relationship of the different species within the genus *Enterococcus* available in the GenBank public database. The phylogenetic tree was constructed based on the 16S rRNA region of 23 *Enterococcus* strains extracted from GenBank with one strain *Lactobacillus plantarum* WCFS1 as an outgroup (Fig. 1). The 16S rRNA sequences were aligned using CLUSTAL W [6] and phylogenetic inferences were obtained using maximum-likelihood method in MEGA (Molecular Evolutionary Genetic Analysis) software 6.0 package [6]. The significance of the branching patterns was evaluated through bootstrap analysis of 1000 replicates.

## Nucleotide sequence accession number

This Whole Genome Shotgun project has been deposited at DDBJ/ENA/GenBank under the accession JAABOR000000000. The version described in this paper is version JAABOR010000000.

## Declaration of Competing Interest

None.

## Appendix Supplementary materials

Image, application 1
